# Committing to quantum resistance: a slow defence for Bitcoin against a fast quantum computing attack

**DOI:** 10.1098/rsos.180410

**Published:** 2018-06-20

**Authors:** I. Stewart, D. Ilie, A. Zamyatin, S. Werner, M. F. Torshizi, W. J. Knottenbelt

**Affiliations:** 1 Centre for Cryptocurrency Research and Engineering, Imperial College London, London, UK; 2 SBA Research, 1, Vienna, Austria

**Keywords:** bitcoin, blockchain, quantum computing, quantum resistance, Elliptic Curve Digital Signature Algorithm

## Abstract

Quantum computers are expected to have a dramatic impact on numerous fields due to their anticipated ability to solve classes of mathematical problems much more efficiently than their classical counterparts. This particularly applies to domains involving integer factorization and discrete logarithms, such as public key cryptography. In this paper, we consider the threats a quantum-capable adversary could impose on Bitcoin, which currently uses the Elliptic Curve Digital Signature Algorithm (ECDSA) to sign transactions. We then propose a simple but slow commit–delay–reveal protocol, which allows users to securely move their funds from old (non-quantum-resistant) outputs to those adhering to a quantum-resistant digital signature scheme. The transition protocol functions even if ECDSA has already been compromised. While our scheme requires modifications to the Bitcoin protocol, these can be implemented as a soft fork.

## 1. Introduction

Bitcoin is a decentralized digital currency system, which was introduced by the pseudonymous Satoshi Nakamoto [1]. It leverages a peer-to-peer distributed network characterized by the lack of a central authority governing the state of transactions. Each consensus participant maintains a list of all historic transactions, grouped together in blocks, in a distributed public ledger called the blockchain.

Blocks are chained together via the hashes of their predecessors, thereby providing strong guarantees for the immutability of the transaction history. Agreement on the current state of the system in the dynamically changing and pseudonymous set of participants is achieved by requiring nodes to solve difficult cryptographic puzzles, known as proof-of-work (PoW). Consensus participants are known as miners and upon finding a valid solution to the PoW puzzle, they are rewarded with new units of the underlying cryptocurrency and fees associated with the transactions included in the respective block.

Even though quantum computers (QCs) have been studied theoretically for about 40 years, relatively recent breakthroughs have placed the idea in the public eye once again. One such breakthrough, with a direct impact on Bitcoin’s security, is Peter Shor’s polynomial time quantum algorithm [2] that can, in its subsequently generalized form, break ECDSA. While more players enter this growing research area, it appears increasingly likely that powerful QCs will emerge in the near future. Although the early generations of QCs do not have enough qubits to solve problems large enough to affect Bitcoin, different alternatives for the architecture of QCs are being considered, tested and implemented [3–5] so a sudden improvement in the approach might lead to a powerful QC appearing virtually overnight.

In this paper, we provide an overview of the potential impacts the emergence of QCs could have on Bitcoin. As such, we describe how a quantum-capable adversary (QCA) is in the position of stealing funds from users who have revealed their public keys. Consequently, we propose a commit–delay–reveal protocol for the secure transition from Bitcoin’s current signature scheme to a quantum-resistant signature scheme, applicable even if ECDSA has already been compromised. In contrast to existing proposals, we emphasize the necessity of a substantial delay phase to provide sufficient protection against accidental and, especially, adversarial chain reorganization. We assume that the Bitcoin community has agreed on and deployed a quantum-resistant signature scheme, either as a measure of precaution or as a reaction to the appearance of a (fast) QCA. Independent of quantum computing, our protocol can be generally applied to react to the appearance of vulnerabilities rooted in Bitcoin’s public key cryptography. The transition can be implemented as a soft fork using a similar approach as, for example, SegWit [6].

The remainder of this paper is organized as follows. In §2, we outline the workings of Bitcoin and provides an introduction on quantum computing. In §3, we examine the threats a QCA could pose for Bitcoin. In §4, we propose a protocol for the transition from Bitcoin’s current signature scheme to a quantum-resistant one, while discussing the implementation details in §5. We discuss a related work in §6 and conclude our paper in §7.

## 2. Background

In the following sections, we provide relevant background on the workings of Bitcoin, its underlying cryptographic principles, as well as core quantum computing concepts, relevant for this paper. However, due to space limitations, we do not aspire to provide a complete description and hence recommend readers unfamiliar with these research fields to consult the existing literature, such as [1,7] for Bitcoin and [8,9] for quantum computing.

### 2.1. Bitcoin and blockchain

In Bitcoin, every transaction consists of inputs and outputs.^1^ Each input references some unspent transaction output (UTXO) and provides a spending script (scriptSig) which will be used to authorize the transfer of funds. Each output is secured by a challenge script (scriptPubKey) which must be solved by the spending script of an input that wants to consume the funds. Based on the challenge script, one can distinguish different types of outputs^2^ :

— pay-to-pubkey (P2PK) outputs were used before the concept of an address appeared. The challenge script contains the public key (*p*_*k*_) associated with the secret key (*s*_*k*_). An input wishing to consume such an output has to provide a digital signature of the transaction. If the signature can be verified against *p*_*k*_, this means that it was indeed created by *s*_*k*_, so the transfer of funds is valid.
— pay-to-pubkeyhash (P2PKH) outputs (presented in user interfaces as ‘addresses’) are 160-bit hashes of the public keys [10]. This has the advantage of saving some space as addresses are shorter than public keys. To consume this type of output, an input needs to provide both the public key that hashes to the address and a digital signature that can be verified with the public key.
— pay-to-scripthash (P2SH) outputs (presented to the users as a new type of ‘address’) are the hash of a script in which the user can specify different conditions to be satisfied by the input scriptSig. One use for this type of addresses is to achieve a compact UTXO format for multi-signature transactions.

Hence, a transaction takes some UTXOs as the source of funds and outputs new UTXOs, associated with the same or a different public key.^3^ As part of Bitcoin’s underlying consensus mechanism, termed Nakamoto consensus, a miner is asked to find the header of a block which includes some random input, or nonce, along with entities such as the hash of the previous confirmed block, such that its hash *h* is below a difficulty threshold. The network difficulty is dynamically adjusted every 2016 blocks such that the average block interval is approximately equal to 10 min. As finding a valid nonce is a memoryless process, the best-known strategy for generating a PoW solution is the enumeration of all possible inputs and is therefore very computationally expensive. On the other hand, other nodes of the network can verify the PoW criterion trivially with a single hash.

### 2.2. Elliptic Curve Digital Signature Algorithm

ECDSA is an implementation of the Digital Signature Standard (DSS) based on elliptic curve cryptography (ECC) [11]. The purpose of such signatures is to allow third parties to determine the legitimacy and integrity of a signed message, while the signer cannot reasonably deny the act of signing. In Bitcoin, transactions are digitally signed using ECDSA, thus securing the transfer of ownership of bitcoins [12].

ECC is a form of public-key cryptography that uses the mathematical properties of elliptic curves over finite fields [11]. More specifically, to define an elliptic curve cryptosystem one chooses a curve *C* and a public point *P* on the curve. To generate a pair of keys, one chooses a random number *sk* as the private key and uses elliptic curve point multiplication [11] to multiply the point *P* with itself *sk* times thus obtaining the public key *pk* which is itself another point on *C*. ECDSA or, in general, ECC, relies on the assumption that it is intractable to solve the elliptic curve discrete logarithm problem (ECDLP) [13], which would allow for deducing the private key from the public key. Like integer factorization [14], ECDLP has no known reasonably fast (e.g. polynomial-time) solution on a classical computer [15].

### 2.3. Quantum computing

Quantum computing makes use of various quantum phenomena, such as superposition and entanglement, to represent classical data in a quantum context and to manipulate it in ways that produce interpretable results [16]. Just like the state of classical computers is made of bits, QCs use qubits that have two fundamental (basis) states (0 and 1). However, while the computation is running, the state is a linear combination (superposition) of basis states, each having an associated probability to be measured.

To extract information about the state of a QC, the system is measured collapsing the superposition to one of the possible basis states. This means a QC with *n* qubits can represent internally the whole range of *n*-bit numbers and can perform calculations on all of them simultaneously; however, when measured, the state will collapse to just one of the basis states, thus returning only one of the results to the performed calculation. Instead, quantum algorithms try to make use of the underlying structure of the problem in order to amplify (or otherwise home in on) certain basis states, to increase their probability, and thus to make the result obtained repeatable and conclusive. For some problems, quantum algorithms can yield a significantly improved runtime complexity over their classical equivalents, thus offering a speed-up.

#### 2.3.1. Shor’s algorithm

Shor’s algorithm for integer factoring is a quantum algorithm with a runtime complexity of $O({\left(\log N\right)}^{2}(\log\log N)(\log\log\log N))$ [2], which is exponentially faster than all known classical algorithms. In fact, the integer factorization problem can be reduced to finding the period of *f*(*x*)=*a*^*x*^ mod  *N* where *a* is a random integer and *N* is the number to be factored [17]. The algorithm works by preparing a superposition of basis states where each basis state is formed by concatenating *x* with the value of *f*(*x*). When the qubits that store *f*(*x*) are measured, the superposition will collapse leaving some value *v* on the qubits measured, while the qubits on which *x* was stored will be in a superposition of different *x*’s with *f*(*x*)=*v*. To obtain the period of the function, the remaining algorithm needs to extract the difference between any of the states in the superposition. The quantum Fourier transform circuit can be used to achieve exactly this [17]. Shor’s algorithm drastically weakens the security of some public-key cryptographic systems such as RSA, but Proos and Zalka show how it can be adapted to solve ECDLP with even fewer steps [18], offering a polynomial-time attack against ECDSA [2].

#### 2.3.2. Grover’s algorithm

Grover’s algorithm is another efficient quantum computation. It aims to solve the problem of searching unstructured data by computing with high probability a unique (or very rare) solution *x* for which *f*(*x*) equals *v*, some desired value [19]. The time complexity is $O(\sqrt{N/t})$, where *N* is the size of the domain of *f* and *t* is the number of solutions [20]. The algorithm works by first arranging a superposition of all possible input states, each having equal probability of being measured. Then, it uses some techniques to iteratively increase the probability amplitude of the states that represent the solution [19]. Given *N* and *t*, the number of iterations after which the probability amplitudes of the correct states become maximal can be mathematically computed [20]. In case *t* is unknown, there exists a scheme which will produce a solution in $O(\sqrt{N/t})$ steps [20].

Note that it is not possible to measure the state after each iteration as this would collapse the superposition and the computation would end. Grover’s algorithm is particularly interesting for mining as it theoretically offers a quadratic speed-up when guessing a nonce. However, in practice, it is believed that early generations of QCs will be slower than optimized ASIC miners [21,22].

### 2.4. Post-quantum cryptography

Post-quantum cryptography is a new branch of cryptography interested in a suite of algorithms which are believed to be secure even against attackers equipped with QCs [23]. There have been multiple proposals of cryptographic systems which are not yet broken by quantum computing. Some examples are:

(i) code-based cryptography relies on the intractability of decoding unknown linear error-correcting codes [24]. McEliece used the algebraic properties of Goppa codes and proposed the first such system [25], which took his name;
(ii) hash-based cryptography is based on the security of hash functions which, as mentioned, are not drastically weakened by QCs. Merkle [26] was the first to propose hash-based digital signatures by building on the concept of one-time signature schemes such as Lamport’s signature scheme [27]; and
(iii) lattice-based cryptography is based on the hardness of lattice problems such as approximating the closest vector problem in a lattice [28].

For the purposes of our paper, it is important that the Bitcoin community agrees on and implements an appropriate alternative (or perhaps more than one) to replace ECC as the basis for digital signatures of transactions.

## 3. Bitcoin in a post-quantum world

Given that we assume a powerful QC could appear at any time, where does this leave Bitcoin? As presented in §2, in a post-quantum world, miners could gain an unfair advantage by mining blocks using Grover’s algorithm. This provides a quadratic speed-up in the number of operations compared to a classical computer, which should lead to an increased hashrate. However, current miners use parallel computations on optimized hardware (ASICs) and it is hence difficult to predict if and when QCs will be reliable and fast enough to outperform them. To this end, we assume that early generations of QCs will not be capable of outperforming classical miners in terms of hash rate. Furthermore, once QCs reach a state of development acceptable for mining, a quick adoption among miners can be expected, establishing an equilibrium as the network difficulty adjusts.

In this paper, we do not aim to address potential vulnerabilities rooted in Bitcoin’s PoW but rather to examine the risks QCAs could pose for the embedded transaction processing mechanism. Once efficient QCs with internal states comprising many qubits are implemented, the underlying cryptographic guarantees of Bitcoin can be challenged. As briefly mentioned in §2, an attacker with a QC of about 1500 qubits [18,22] can use Shor’s algorithm to solve the ECDLP and compute an ECDSA private key given the public key, and is thus able to plant fake transactions and perform double-spending attacks. In the following sections, we highlight why Bitcoin users should be concerned about exposing their public keys and describe a potential attack scenario whereby a QCA engages in (live) transaction hijacking.

### 3.1. Public key unveiling

Under the assumption that QCs are being employed for malicious intent by some adversary, previously revealed public keys pose a direct threat to Bitcoin users. As outlined, a QCA is capable of deducing the private key from a formerly revealed public key with little effort. Such a scenario could arise from the following instances of public key unveiling:

(i) Bitcoin transactions with *P*2*PK* UTXOs, as these display the public key in the output of the transaction. As soon as such a transaction has been included in the blockchain, or even just broadcast to the network, a slow QCA can compute the corresponding private key and thereby essentially gain control over the respective funds. An initial analysis of the UTXO set in Bitcoin shows that about 1.77 million BTC fall into this category.^4^ This problem can be mitigated by using, for example, *P*2*PKH* and *P*2*SH* addresses. However, when consuming such an UTXO, the owner of the address must reveal her public key and digital signature in the scriptSig of the respective input. Once this transaction is broadcast to the network for confirmation and inclusion in a block, the attacker can compute the private key from the revealed public key. Furthermore, the attacker could then look for any additional UTXOs associated with the same address and consequently consume them, now that she is in control of the private key. We find that about 3.9 million BTC reside in UTXOs that can be compromised by such attacks. In total, at least 33% of all BTC are currently vulnerable to attacks by a QCA. At the time of writing, this amounts to approximately 50 billion USD.^5^
(ii) Bitcoin users publishing their public key on a Bitcoin fork, e.g. Bitcoin Cash [31] or Bitcoin Gold [32]. As Bitcoin forks share the same transaction history prior to the fork point, such behaviour may allow a QCA to gain control over a user’s Bitcoin funds using the exposed public key. Furthermore, a QCA could then also exert control over funds on the blockchain where the public key was initially obtained.
(iii) Any other revealing of public keys, such as part of signed messages to ensure integrity, in forums, or in payment channels (e.g. Lightning Network [33]).

Regardless of how a public key is revealed, given the presence of a QCA, the owner is at risk of losing control over her funds. Except for P2PK, one can prevent against the aforementioned scenarios (so long as the QCA is slow to deduce a private key) by using addresses only once. Reusing addresses is not recommended, neither by Bitcoin developers nor the community, while numerous studies identifying privacy risks have been conducted [34–38]. Hence, we assume appropriate protective mechanisms are already employed by the majority of Bitcoin users.

### 3.2. Transaction hijacking

We assume that a fast QCA is characterized by the ability to perform (live) transaction hijacking. Thereby an attacker attempts to compute the private key corresponding to a public key revealed in the input of a transaction published to the network and sitting in nodes’ memory pools. Consequently, just like in a double-spending attack [39–42], she creates a conflicting transaction spending the same UTXOs^6^ (or the subset the QCA has gained control over), thus stealing the victim’s funds. As the attacker must not only create, sign and broadcast the conflicting transaction, but also first run Shor’s algorithm to derive the private key, timing is essential for such attacks. Hence, the performance of QCs plays a central role for the success probability of transaction hijacking. Note that this form of transaction hijacking differs from the more conventional notion of double spending as the attacker is the sole beneficiary rather than the original transaction initiator.

We do not discuss the possibility of using Grover’s algorithm to retrieve the public key from an address here, as the achieved speed-up is merely quadratic and can be mitigated by increasing the key size [21]. However, an attacker could potentially use Grover’s algorithm to gain an unfair advantage when mining.

An extension to the described attacks is to combine transaction hijacking with selfish mining strategies [38,43–45]. Assuming the QCA is also a miner, she could employ her computational power to attempt to build up her own secret chain and, when in the lead, selectively publish blocks to cause a reorganization of the public chain. In contrast to traditional selfish mining attacks, the feasibility of such strategies under the presence of a QCA is expected to improve significantly, as the adversary can now also perform transaction hijacking as described above. The prospectively gained revenue consists not only of block rewards and transaction fees, but also of all funds contained in (non-quantum-resistant) UTXOs spent in the overwritten transactions.

## 4. Transition to quantum resistance

In this section, we describe a scheme that allows a secure transition from Bitcoin’s current signature scheme to a quantum-resistant one. We assume a quantum-resistant signature scheme has already been agreed upon by the community, and deployed as a protocol update in Bitcoin. However, it is presumably unreasonable to hope that all Bitcoin users will have moved their coins from non-quantum-resistant to quantum-resistant outputs; inevitably, some people (perhaps even a majority) will still be storing some or all of their currency units in non-quantum-resistant outputs, especially the most popular P2PKH. The protocol described in the following sections is designed to allow such users to transition securely, if rather slowly, to quantum-resistant outputs even in the presence of a fast QCA. It is based on a simple commit–delay–reveal mechanism with a long security delay, and can be deployed in Bitcoin using a soft fork. Bitcoin-specific implementation details as well as discussion on parametrization and necessary data structures are considered separately in §5. Note that once the protocol is deployed, classic ECDSA signatures will no longer be accepted and clients will only be allowed to spend UTXOs based on the previously introduced quantum-resistant signature scheme or the transition scheme described in this paper. Furthermore, if one uses an old client and spends from a non-quantum-resistant public key, the respective funds will be lost, as the public key is revealed, and no protective mechanism can be applied effectively.

### 4.1. Protocol overview

**Figure 1.**
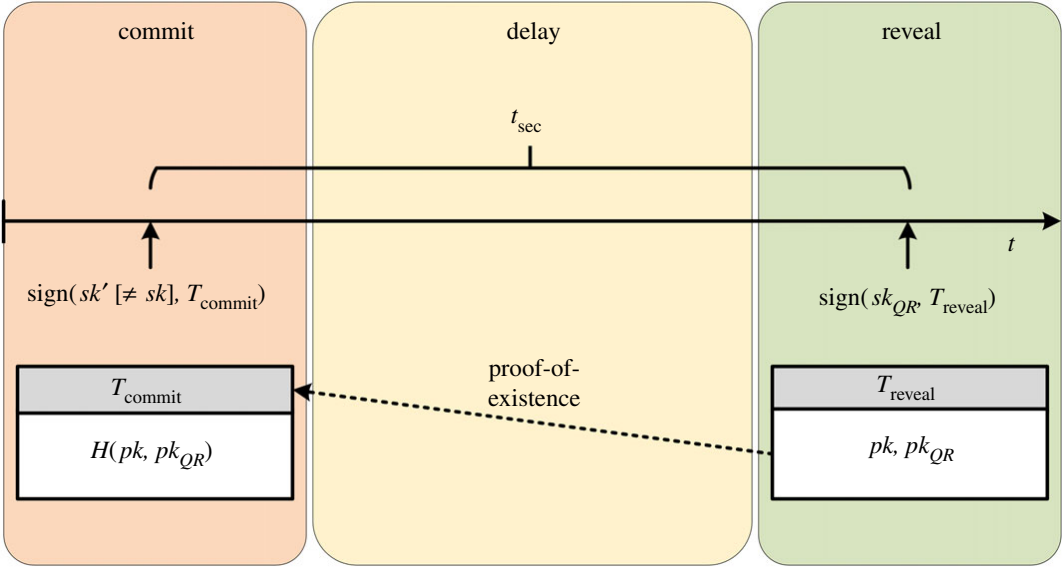
Simplified visualization of the commit–delay–reveal transition scheme.

Assume a user, Bob, is in possession of units of Bitcoin stored in a non-quantum-resistant output, the public key of which has not yet been revealed, i.e. funded by an unspent P2PKH or P2SH output.^7^ We shall denote Bob’s public key as **pk** and the corresponding secret key as **sk**. Furthermore, assume Bob has already generated a quantum-resistant keypair (**pk*_*QR*_*,**sk*_*QR*_*), which will be used as a surrogate for his current keypair (during any future spending) as part of the transition. To convince the network, he is the rightful controller of both keypairs and this way regain the ability to safely spend the funds at a future date in any way he pleases (e.g. to pay user Carol), Bob publishes a commitment *H*(**pk**|**pk*_*QR*_*), i.e. the hash of his concatenated public keys, and leaves the funds on *pk* untouched for a sufficiently long security period *t*_*sec*_. Once the period has passed, Bob creates a second transaction *T*_*reveal*_ signed by **sk*_*QR*_* which consumes all UTXOs attributed to (*pk*,*sk*) and reveals both public keys **pk** and **pk*_*QR*_*, proving to the network that he is the controller of both keypairs and signalling the transition of funds. We illustrate this process in figure 1 and detail each step in the following paragraphs.

### 4.2. Commit

As a first step, to mark the commitment of the funds in (*pk*,*sk*), Bob publishes the hash of both public keys *pk* and *pk*_*QR*_ concatenated: *H*(**pk** | **pk*_*QR*_*). This is achieved by creating a transaction *T*_*commit*_, which includes the hash commitment as an output. In Bitcoin, this can be achieved, for example, by using the OP_RETURN opcode, which allows us to store up to 80 bytes of arbitrary data in a transaction [46].^8^

Note that as Bob cannot spend any non-quantum-resistant coins to fund the creation of the OP_RETURN, he will have to either already possess, or acquire through trade, some quantum-resistant currency units—sufficient to fund the creation of an OP_RETURN on the blockchain.

### 4.3. Delay

After publishing the hash commitment, Bob leaves the funds in (*pk*,*sk*) untouched for a sufficiently long security period *t*_*sec*_. Any further attempted use of this keypair, which would fail in accordance with the new protocol rules, puts Bob’s funds at risk of theft. A long delay is necessary to ensure no blockchain reorganization could have occurred accidentally or have been caused intentionally by an adversary. While the specific choice of delay may be subject to follow-up scientific work and discussion in the community, we propose an initial period of six months. A more detailed discussion is provided in §5.

### 4.4. Reveal

Once the security period has elapsed, Bob proceeds to safely spend the coins to any destination(s) he pleases, by revealing his public keys *pk* and *pk*_*QR*_, proving to the network he is the rightful controller of both keypairs. To this end, Bob creates a transaction *T*_*reveal*_ signed by the secret key **sk*_*QR*_* of the new quantum-resistant keypair, which consumes the UTXOs of (*pk*,*sk*) and in which he

(i) gives his ‘old’ non-quantum-resistant public key **pk**,
(ii) gives the public key of the new quantum-resistant keypair **pk*_*QR*_* ,
(iii) reveals (via Merkle-tree proof) that he has published *H*(**pk** | **pk*_*QR*_*) in a transaction older than the security period *t*_*sec*_, and
(iv) provides a quantum-resistant signature of the transaction against **pk*_*QR*_*.

Miners, adhering to the new protocol rules, will then be able to verify the funds that have been committed for a sufficient period to require a new quantum-resistant public key for their eventual spending. Hence, Bob will be allowed to spend his funds by providing a valid signature against his new quantum-resistant public key. Unupgraded consensus participants will simply believe *T*_*reveal*_ is a normal transaction consuming the UTXOs of (*pk*,*sk*). The necessary implementation specifics are provided in §5. As a result, the protocol update *P*→*P*′ can be deployed as a soft fork, as the set of blocks valid under new rules *P*′ is a proper subset of blocks valid under current Bitcoin rules *P*, i.e. *P*′⊂*P*.

## 5. Discussion

In this section, we discuss selected implementation details of the introduced commit–delay–reveal transition scheme, including the choice of the delay period and the structure of the commit and reveal transactions.

### 5.1. Necessity for a long delay phase

The correct choice of the security period *t*_*sec*_, used as protection against accidental and adversarial chain reorganizations, has a significant impact on the security properties of the proposed transition protocol. In contrast to previous proposals and discussions [47–50], we emphasize the necessity of a sufficiently long delay phase, substantially longer than the standard confirmation period of approximately six blocks in Bitcoin. While the exact duration of *t*_*sec*_ may be subject to future discussion, we propose to require hash commitments to be older than six months, i.e. the UTXOs used as input to *T*_*reveal*_ must remain unspent during this period.

As explained in §3, we assume that the feasibility of block reorganization attacks, such as 51% attacks or selfish mining attacks requiring a smaller fraction of the overall computational power, is significantly increased for QCAs. In contrast to traditional reorganization attacks, the prospective gains in this scenario are not only composed of block rewards and transaction fees but also include any funds whose public keys have been revealed in one of the blocks overridden by the attacker. Hence, relying on a short security period of a few blocks (or no delay at all) provides insufficient protection against chain reorganizations in the presence of a QCA.

We note that in theory an adversary controlling a significant portion of the overall computational power could successfully rewind the chain further than *t*_*sec*_, thereby altering the transaction history, and attempt to steal funds from all non-quantum-resistant outputs which were spent from during this period. However, we argue a fork overriding the block history of such substantial period as six months would be classified as a catastrophic failure of the system, forcing out-of-band measures to be undertaken by the majority of honest consensus participants. Specifically, we assume clients and miners will have incentive to manually reject the conflicting branch of the attacker.^9^

**Figure 2.**
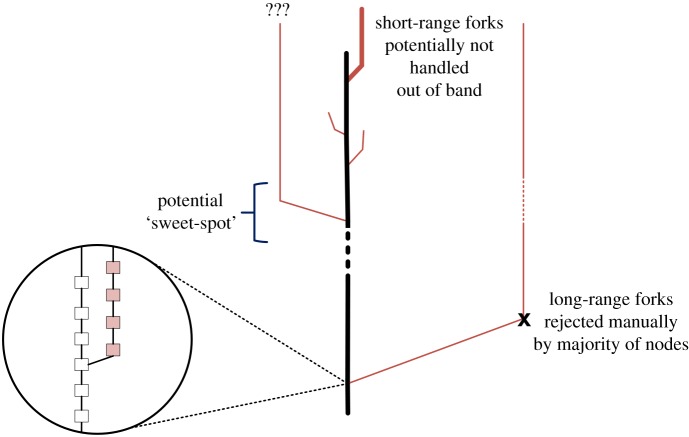
While long-range forks are expected to be manually rejected by the majority of nodes, this may not be possible with short-range chain-splits due to the limited time frame. There may exist a ‘sweet-spot’ which causes a dispute whether to accept or reject the conflicting branch, destabilizing or even permanently splitting the network to the benefit of the adversary (red).

However, by intuitive continuity arguments, there must exist a point between short- and long-ranged attacks, where the community is unable to find even out-of-band consensus on how to proceed, i.e. whether to perform a manual invalidation (override of attacker’s fork) soft fork or accept the conflicting branch of the adversary, as visualized in Figure 2. While under different circumstances, similar disputes have been observed in other cryptocurrencies and have led to permanent chain splits, as in the case of Ethereum [52] and Ethereum Classic [53]. Hence, a QCA may have the incentive to attempt to exploit this ‘sweet-spot’ to her advantage, as a destabilization or split of the chain could yield a higher success probability of an attack.

By implementing a long delay phase, sufficient to trigger out-of-band actions in case a longer fork is created by an adversary, the probability of a malicious chain reorganization interfering with the transition protocol can be minimized.

#### 5.1.1. Arguments against parametrization by users

Instead of defining the delay phase *t*_*sec*_ as part of the consensus rules, a naive approach would be to allow each user to declare their own security period. However, we emphasize the necessity of a global fixed delay phase, as allowing individual parametrization by users leaves the transition scheme vulnerable to attacks, as described in the following.

##### 5.1.1.1. Declare *t*_*sec*_ during Reveal

A straightforward approach would be for users to declare their own security period *t*_*sec*_ during the reveal phase, i.e. in *T*_*reveal*_. The problem with this approach, however, is that a QCA can then derive the user’s secret key *sk* from the now revealed public key *pk*. The attacker will then attempt to revert the public chain so that *T*_*reveal*_ is no longer included in the blockchain, and create a new hash commitment *H*(*pk*,*pk*′_*QR*_) for her own quantum-resistant public key *pk*′_*QR*_ by publishing *T*_*commit*_′. After waiting for a minimal period to be sure, the majority of consensus participants have accepted the attacker’s chain, she moves on to reveal the victim’s (non-quantum-resistant) and her own (quantum-resistant) public key in *T*_*reveal*_′, declaring a very short security period. As a result, the victim’s funds in (*pk*,*sk*) will be transferred to the attacker’s control. Note that it is sufficient for the adversary to be able to revert only a few blocks for this attack to be successful.

##### 5.1.1.2. Declare *t*_*sec*_ during Commit:

A possible way of mitigating the attack described above is to require users to declare *t*_*sec*_ as part of the hash commitment and employ a ‘first-seen’ rule, i.e. only consider the first commitment included in the blockchain for each public key as valid. However, at reveal time, validators (consensus participants) need to be able to verify that the linked commitment is indeed the first commitment associated with this public key. For this, users are required to make their hash commitments publicly verifiable, i.e. include a hash of the public key in *T*_*commit*_. While this approach prevents an attacker from overriding a user’s reveal transaction, it also enables griefing (overriding the commit transaction). As such, an adversary could easily listen for commit transactions, grab the hash of the public key used in them and publish fake commitments before the original is included in the blockchain, thus preventing the transition of funds altogether.

### 5.2. Structure of the hash commitment

During the commit phase of the protocol, a transaction *T*_*commit*_ containing the hash commitment for *pk* and *pk*_*QR*_ is created. As mentioned, the Bitcoin OP_RETURN script operation allows up to 80 bytes of arbitrary data to be pushed onto the stack [46], which is sufficient to, for example, persist a SHA-256 hash. However, there may also be alternatives to this way of publishing the hash commitment.

The exact format of the hash commitment, having little impact on the introduced transition protocol, is expected to be subject to an open discussion in the community. For simplification, we propose to use the concatenation of the public keys *pk* and *pk*_*QR*_ as input to the hash function. This, however, assumes that agreement on the quantum-resistant signature scheme has been reached beforehand.

### 5.3. Reveal structure and backward compatibility

During the reveal phase of the transition protocol, users must prove to the network they are the rightful controllers of the non-quantum-resistant keypair (*pk*,*sk*) and the quantum-resistant keypair (*pk*_*QR*_,*sk*_*QR*_), and provide evidence that there exists a hash commitment for the public keys of these keypairs older than the security period *t*_*sec*_. Note that *T*_*reveal*_ is signed with *sk*_*QR*_, thus proving control of the quantum-resistant keypair. The commitment proof is achieved by providing a SPV (simplified payment verification) proof [54,55], i.e. including the path to *T*_*commit*_ in the Merkle tree transaction structure of the respective block, dating back *t*_*sec*_ or more, in the reveal transaction *T*_*reveal*_. To enable the deployment of the transition scheme as a soft fork, i.e. without requiring a permanent split of the blockchain, we propose a scheme similar to that used in SegWit [6]. As such, the data witnessing the new rules are being obeyed are held in a segregated area, termed QRWitness, which new clients receive and check but old clients remain oblivious to. To make sure the witness structure is committed to by (the header of) the block it is contained in, the root of a Merkle tree consisting of all QRWitness-es is inserted in the respective coinbase transaction. While the original transaction txid remains the same as before, a new qrtxid is defined as the double SHA256 hash over the traditional transaction format and the QRWitness. Thereby, a possible format for QRWitness could be the following:

<oldPubkey><pubkeyQR><merklepath><signatureQR>

where oldPubkey denotes the non-quantum-resistant public key *pk*, pubkeyQR is the quantum-resistant public key *pk*_*QR*_, merklepath represents the path to the hash of the *T*_*commit*_ transaction and signatureQR denotes the signature of the traditional transaction format using *sk*_*QR*_.

To achieve backward compatibility, the scriptSig field remains such that it satisfies the consensus rules of old clients, e.g. the non-quantum-resistant signature and the corresponding public key. This way, just like SegWit, our transition protocol can be deployed as a soft fork in Bitcoin. Note however, that as usual with soft fork attempts, if the majority of the mining power does not upgrade and continues to accept transactions spending non-quantum-resistant outputs without adhering to the commit–delay–reveal structure, the soft fork will cause a potentially permanent split of the blockchain.

The extension to more diverse challenge scripts than are covered by the usual address types should now be clear; namely, provide in the QRWitness as many instances of <oldPubkey><pubkeyQR><merklepath><signatureQR> as are necessary, i.e. one for each act of checking a non-quantum-resistant signature under the old rules. Thus, all patterns of ECDSA signature verification (including multi-sig and puzzle-type scripts) will require the appropriate associated post-quantum signature check(s) as a matter of course.

## 6. Related work

The possibility of QCs emerging in the near future is increasingly appreciated by members of the Bitcoin community and hence a number of approaches to make Bitcoin resilient against QCAs have recently been discussed.

As discussed briefly in §4, a first step towards maintaining Bitcoin’s security properties in a post-quantum world is seen in replacing ECDSA with a signature scheme believed to be quantum resistant, which can be implemented on classical computers [56–58]. Other proposals rely on quantum hardware to exploit quantum effects to guarantee the security of the cryptocurrency against QCAs [59–61]. An alternative research direction focuses on identifying alternatives to PoW, as a countermeasure to possible unfair advantages in mining through Grover’s algorithm [62,63].

However, the aforementioned works do not consider the issue our paper is most interested in, i.e. transitioning to post-quantum Bitcoin in the presence of an already-fast QCA. While our methods were developed independently, we provide an overview of relevant discussions, papers and articles we have become aware of, which attempt to solve this problem.

A first brief public mention of a scheme transitioning Bitcoin to quantum resistance is made by Back, referring to an informal proposal by Lau [47,64–66]. The discussed approach leverages on a two-phase commit mechanism, similar to the one described in this paper, i.e. users commit *H*(*pk*,*pk*_*QR*_) in the OP_RETURN field of a transaction and, after waiting for confirmations, create a transaction which reveals (*pk*,*pk*_*QR*_). However, the scheme is not discussed in detail and the requirements for the security delay *t*_*sec*_ between commit and reveal phase, necessary to mitigate transaction reordering attacks by QCAs potentially benefiting from Grover’s algorithm, are left open.

An alternative scheme described by Ruffing in the Bitcoin-dev mailing list [48–50] requires users to create the transaction spending the non-quantum-resistant UTXOs in advance, as it must be part of the commitment. The transaction is thereby encrypted with a symmetric key *k* derived from the challenge chal used to generate the address associated with the transitioned UTXOs. Once the commitment transaction is confirmed, the user finalizes the transition by publishing chal, which allows the network to derive *k* and decrypt the spending transaction. However, similar to Back’s proposal, the scheme does not discuss the duration of *t*_*sec*_. Specifically, while it appears feasible for a user to predict the target of the spending transaction in case *t*_*sec*_ is equal to a few blocks, this does not necessarily hold if circumstances require longer delays.

Fawkescoin [67] is a cryptocurrency which relies only on secure hash functions, avoiding the use of asymmetric cryptography. While not aiming at transitioning to a quantum-resistant signature scheme, it introduces a commit–reveal scheme to move funds from an owner (*O*_*X*_) of secret *X* to the owner (*O*_*Y*_) of a secret *Y* . Thereby, *O*_*Y*_ sends a hash *H*(*Y*) of her secret to *O*_*X*_, who proceeds to include a hash commitment *H*(*X*,*H*(*Y*)) in the underlying blockchain, thereby guaranteeing to send the funds linked to *X* to whoever can provide the secret *Y* . After a pre-defined confirmation period, *O*_*X*_ publishes the input to the hash commitment (*X*,*H*(*Y*)), revealing *X* to the network. Consequently, (only) *O*_*Y*_ can now spend the funds linked to *X*, using her secret *Y* . Note in Fawkescoin, users must know the destination of the transfer at the time of commitment, while our scheme is flexible and imposes no such requirements by construction.

## 7. Conclusion

In the light of the emerging threat of QCAs in Bitcoin, we have outlined how Bitcoin could become subject to theft of funds rooted in the exposure of public keys. Thus, we have proposed a commit–delay–reveal scheme to allow for the secure transition to a quantum-resistant address scheme in Bitcoin, the underlying protocol modifications for which can be implemented as a soft fork. For the security of the transition scheme we emphasize the need for a sufficiently long delay period and propose an initial period of six months in order to prevent possible blockchain reorganization. The proposed time frame should suffice for allowing honest clients and miners to reach consensus on manually rejecting long-range forks that exceed the delay period. However, we suggest that by intuitive continuity arguments there must exist some length of chain-rewind time where the community would be indecisive on how to proceed given that a conflicting branch created by an adversary exists. Hence, we note that the optimal duration of the delay period may be subject to future discussion and analysis.

## Footnotes

1 With exception of the first transaction in each block in which new units of Bitcoin are released, termed coinbase transaction.

2 There are actually many more types of challenge scripts and in §5 we briefly discuss how to make our transition protocol general enough to secure them all.

3 Using a different public key is recommended for security and privacy reasons.

4 The empirical analysis was conducted using BlockSci [29] and considers data up to Bitcoin block 514877 (24 March 2018).

5 Calculated using a BTC/USD exchange rate of $9369 [30].

6 Possibly with a higher fee to incentivise inclusion in the blockchain over the victim’s transaction

7 Our protocol actually caters for spending any number of such UTXOs in one transaction, but for simplicity we will consider here the spending of only one.

8 There may be other ways; we leave it up to the developers and/or the community to choose an appropriate format.

9 Note that this does not require any changes to the reference client implementation, as Bitcoin’s JSON-RPC API provides a invalidateblock call, which permanently marks a specific block as invalid, as if it had violated a consensus rule [51]. (There are of course many other clients available, some of which may require code changes to manually reject a branch.)

## Data accessibility

This article has no additional data.
